# Bayesian Optimization Based on K-Optimality

**DOI:** 10.3390/e20080594

**Published:** 2018-08-09

**Authors:** Liang Yan, Xiaojun Duan, Bowen Liu, Jin Xu

**Affiliations:** College of Liberal Arts and Sciences, National University of Defense Technology, Changsha 410000, China

**Keywords:** design of experiments, K-optimal design, gaussian processes, bayesian optimization

## Abstract

Bayesian optimization (BO) based on the Gaussian process (GP) surrogate model has attracted extensive attention in the field of optimization and design of experiments (DoE). It usually faces two problems: the unstable GP prediction due to the ill-conditioned Gram matrix of the kernel and the difficulty of determining the trade-off parameter between exploitation and exploration. To solve these problems, we investigate the K-optimality, aiming at minimizing the condition number. Firstly, the Sequentially Bayesian K-optimal design (SBKO) is proposed to ensure the stability of the GP prediction, where the K-optimality is given as the acquisition function. We show that the SBKO reduces the integrated posterior variance and maximizes the hyper-parameters’ information gain simultaneously. Secondly, a K-optimal enhanced Bayesian Optimization (KO-BO) approach is given for the optimization problems, where the K-optimality is used to define the trade-off balance parameters which can be output automatically. Specifically, we focus our study on the K-optimal enhanced Expected Improvement algorithm (KO-EI). Numerical examples show that the SBKO generally outperforms the Monte Carlo, Latin hypercube sampling, and sequential DoE approaches by maximizing the posterior variance with the highest precision of prediction. Furthermore, the study of the optimization problem shows that the KO-EI method beats the classical EI method due to its higher convergence rate and smaller variance.

## 1. Introduction

Computer simulations are widely used to reproduce the behaviour of systems [1,2] through which their performance can be estimated. Usually surrogate models are introduced to represent the physical realities which can be computationally expensive and are difficult to obtain analytical solutions for. In general, *f* is denoted as a response function of the real system with input $x\in X\subseteq R^{D}$ and observation y∈R which follows the form below:

(1) $$\begin{array}{c} y=f(x)+\epsilon . \end{array}$$

Both x and ϵ are regarded as random parameters. Given *N* samples ($X=\left\{X_{1},\ldots ,X_{N}\right\}\in X^{N},X_{i}\in X$) and corresponding observations ($Y\in R^{N}$), the surrogate models can be built to approximate f(x) along with its statistics. The problem of proposing proper X is known as the Design of Experiments (DoE) and it was developed with various mathematical theories. Basically, DoE methods can be categorized as model-free and model-oriented.

The Monte Carlo (MC) [3,4] method is a typical model-free DoE technique and has been widely used in applications. The main advantage of MC method is its simplicity in implementation. However, it converges at a rate of $O(N^{-1/2})$. As a consequence, a large *N* is usually needed to obtain an acceptable result and it is unsuitable for large scale high dimensional problems. A widely used way to accelerate the MC method is the quasi-MC technique [5], for example, quasi-MC based on the Sobol set and Holton set. Another way to substitute the MC method is the Latin Hypercube Sampling (LHS) technique [6] which can generate a near-random sample from a multidimensional distribution with even probability in a pre-defined grid, which ensures the sample is representative of the real variability.

In the context of surrogate models, given a parametric or non-parametric model, we aim to estimate the corresponding parameters or hyper-parameters to achieve the most accurate model. The model-oriented DoE is obtained via some pre-specified criteria. In parameter estimation problems, a popular approach is to consider information-based criteria [7]. An A-optimal design minimizes the trace of the inverse of the Fisher information matrix (FIM) on the unknown parameters, whereas E-, T-, and D-optimal designs maximize the smallest eigenvalue, the trace, and the determinant of the FIM. In the Bayesian framework, the Markov Chain Monte Carlo (MCMC) method [8] is an adaptive DoE technique which utilizes the prior and posterior information; hence, it can focus on points with more important information. The main shortage of MCMC is that it has difficulty determining the acceptance-rejection rate, and it sometimes seems cumbersome because of the long term burn-in period.

Nowadays, more efforts have been devoted to sequential sampling strategies with non-parametric Gaussian process (GP) models [9,10,11]. The main idea behind those methods is to minimize the times required to call the original system which can be computationally expensive. A learning criterion should be given in prior to obtain samples sequentially. B. Echard et al. [12] have proposed the active learning reliability method which combines the Kriging and Monte Carlo simulation methods (AK-MCS) to iteratively assess the reliability in a more efficient way. Similarly, for continuous functions, Bayesian optimization (BO) [10], despite being designed to solve the optimization problem, also collects samples adaptively. The learning criterion is known as the acquisition function in the BO field. One can optimize the expected improvement (EI) or the probability improvement (PI) over the current best result or the lower/upper confidence bound (LCB/UCB) to decide the next point to be sampled. Unlike the A(E,T,D)-optimal designs which decide the DoE in one step, sequential sampling strategies utilize the information from the observations, hence producing more reliable and accurate results for our research goals.

We note that two main obstacles of the BO exist: first, the optimization of hyper-parameters and the inference of Gaussian processes may fail when the covariance in the Gram matrix of the kernel with respect to current DoE X is ill-conditioned; second, it is usually difficult to determine the trade-off parameter between exploration and exploitation, i.e., local optimization or global search. To solve the first problem, considering a similar situation where a parametric regression problem becomes unstable when the condition number of its design matrix is large, the state-of-the-art K-optimal design [13] which optimizes the condition number could be a reasonable choice. In this paper, a new BO approach is proposed with the condition number of the Gram matrix being introduced as an acquisition function, namely, the Sequentially Bayesian K-optimal design (SBKO). We show that the SBKO actually evolves towards the direction of reducing the integrated posterior variance as well as the direction of maximizing the KL divergence between the prior and posterior distributions of hyper-parameters. No extra parameter is needed to balance the exploration and exploitation, because the SBKO generally tends to fill the whole design space; hence, it is suitable for global search tasks such as approximation and prediction. To solve the second problem, the property of K-optimality can be also used to modify the trade-off parameter, based on the idea that those points leading to smaller condition numbers should be explored. We combine the K-optimality and the classical BO criterion to propose the K-optimal enhanced BO (KO-BO) method. The trade-off parameters are computed automatically according to changes in the condition number brought by the associate points. Compared with the classical BO methods, the KO-BO method is more flexible in determining the trade-off parameter, and it implicitly ensures the stability of the GP model.

The paper is organized as follows. We review Gaussian process regression in Section 2 along with the K-optimal criterion. Our main method and algorithm are in Section 3. At the beginning of Section 3, we present the corresponding acquisition function to incorporate the K-optimal design with the BO framework, i.e., the Sequentially Bayesian K-optimal design. Secondly, we show the connections of our method with the methods that focus on minimizing the integrated posterior variance and maximizing the information gain of the inference respectively. At the end of Section 3, we propose the K-optimal enhanced Bayesian optimization algorithms to solve the optimization problem. The experimental results are presented in Section 4, and the conclusions follow in Section 5.

## 2. Brief Review

In this section, we briefly review the general procedure of Gaussian processes and Bayesian optimization approaches, before discussing our novel contributions in Section 3.

### 2.1. Gaussian Processes

Firstly, we assume that a Gaussian prior is set over function *f*, i.e., f∼GP(0,k), where the mean function is set to be 0 and k:X×X→R is the kernel function. Given DoE X and the corresponding observations (*Y*), we have the likelihood as follows:

(2) $$Y|X∼N(0,K+\sigma^{2}I).$$

The predictions (f(x)) at a new point (x∈X) can be sampled from the posterior estimation:

(3) $$\begin{array}{ccc} f(x)|x,X,Y & ∼ & \;N(m(x),v(x)),\\ m(x) & = & \;K_{x}^{T}K_{\sigma }^{-1}Y,\\ v(x) & = & \;K_{\mathrm{xx}}-K_{x}^{T}K_{\sigma }^{-1}K_{x}, \end{array}$$

where $K_{\sigma }=K+\sigma^{2}I$ with $K={\left[k\left(X_{i},X_{j}\right)\right]}_{ij}$ denoting the N×N matrix of the covariances at all pairs of training points, and $K_{x}={\left[k\left(X_{i},x\right)\right]}_{i}$, $K_{\mathrm{xx}}=k\left(x,x\right)$ are defined similarly. It is worth noting that the posterior mean estimation (m(x)) is just a combination of observations (*Y*), and the posterior variance is actually independent of *Y*—it is mainly determined by the kernel function.

### 2.2. Bayesian Optimization

There are two main aspects in Bayesian optimization. Firstly, the prior assumption about the surrogate model, i.e., Gaussian processes in this paper discussed in previous subsection, must be selected. Secondly, an acquisition function must be constructed based on the model posterior, which can be used to sample the “best” point sequentially. We denote the acquisition function as $a_{\mathrm{ac}}\left(x\right):X\to R^{+}$, and then the next entry of the expensive original system is determined by an optimization problem, for instance, $x_{\mathrm{next}}=\arg\max_{x\in X}a_{\mathrm{ac}}\left(x\right)$. In general, the shape of the acquisition function depends on the previous learning results, i.e., the mean and variance of the GP prediction. As mentioned in the introduction, several popular acquisition functions exist. The best value is denoted as $\left\{x_{\mathrm{best}}^{t}=\arg\min_{x\in X^{t}}f\left(x\right),y_{\mathrm{best}}^{t}=f\left(x_{\mathrm{best}}^{t}\right)\right\}$, and $X^{t}$ is the DoE at iteration *t*. Thus, we have the PI, EI, and LCB acquisition functions as follows:

**Probability Improvement (PI):** The idea of the PI method is to maximize the probability of improving the current best value. Under the GP assumption, it has the following form:
  (4) $$a_{\mathrm{PI}}\left(x\right)=P\left[m\left(x\right)<y_{\mathrm{best}}^{t}\right]=\Phi \left(\gamma \left(x\right)\right),\;\gamma \left(x\right)=\frac{y_{\mathrm{best}}^{t}-m\left(x\right)}{v(x)},$$
  where Φ(·) is the cumulative distribution function of the standard normal distribution.
**Expected Improvement (EI):** Alternatively, we can choose to maximize the expected improvement over the best current value. The explicit mathematical expression is given as follows:
  (5) $$a_{\mathrm{EI}}\left(x\right)=E\left[{\left(y_{\mathrm{best}}^{t}-m\left(x\right)\right)}^{+}\right].$$
**Lower Confidence Bound (LCB):** The LCB criterion aims to minimize the regrets over the course of their optimization, and it has the following form:
  (6) $$a_{\mathrm{LCB}}\left(x\right)=-m\left(x\right)+\xi_{t}v\left(x\right),$$
  where $\xi_{t}$ is a constant to trade off the exploration and exploitation.

### 2.3. K-optimal Design

The K-optimal design is based on the idea of finding a specific set of support points which results in the smallest condition number of the information matrix. The *p*-th order polynomial regression model is investigated, and a theoretical symmetry DoE in the space [−1,1] was given in the original paper of Ye [13], where the boundary is usually included. Sándor Baran [14] extended the K-optimal to the correlated processes, i.e., Ornstein–Uhlenbeck processes, in his research. The simulation results in reference [14] show the superiority of restricted K-optimal designs for large covariance parameter values. So, the K-optimal design has potential application in deriving stable and accurate approximations. We embedded the K-optimality into the Bayesian optimization framework, where a sequentially K-optimal design was sampled iteratively. The main methodology and corresponding discussions are given in the next section.

## 3. Methodology

We restate that our main goal was to choose an optimal design from the predefined input domain which is appropriate for inferring the model in the Bayesian framework. The Gaussian processes was chosen as the model, while the K-optimality was taken into consideration. As reviewed in Section 2, the performance of the Gaussian processes was generally controlled by the covariance functions, i.e., kernels, which are continuous, positive semi-definite functions. It is notable that an inverse term of $K_{\sigma }$ exists in Equation (3). When the collected samples are close enough, it will lead to potential failure to calculate $K_{\sigma }^{-1}$ as well as the inference of the Gaussian processes, although a nugget term $\sigma^{2}I$ was added.

In this work, we focused on an experimental design that ensured the correctness and accurateness of Bayesian inference. If the condition number of *K* in Equation (3) is bounded by a relative small constant, then the inference of Gaussian processes can be always achieved. The Sequentially Bayesian K-optimal design (SBKO) was then proposed which is straightforward and simple to present. Like the classical BO methods, the acquisition function is given as $a_{K}=\kappa \left(K_{\sigma }\left(x;\theta \right)\right)$, where κ(·) stands for the condition number and $K_{\sigma }\left(x;\theta \right)$ is the updated covariance matrix, with θ being the hyper-parameters of the kernel function. The term θ can be omitted in the following sections without causing any misunderstanding. Hence, $K_{\sigma }\left(x\right)$ is defined as follows, and the next point ($x_{\mathrm{next}}$) is sampled by solving the optimization problem:

(7) $$\begin{array}{cc} x_{\mathrm{next}} & =\arg\min_{x\in X}a_{K}\left(x\right),\\ K_{\sigma }\left(x\right) & =\left[\begin{array}{cc} K_{\sigma } & K_{x}\\ K_{x}^{T} & K_{\mathrm{xx}}+\sigma^{2} \end{array}\right]. \end{array}$$

There are two main concerns about the minimization of $a_{K}\left(x\right)$. On one hand, the condition number and its optimization problem are not convex. Hence, non-smooth algorithms, such as the DIviding RECTangles (DIRECT) algorithm [15] or the genetic algorithm, are used to solve Equation (7). A few works in the literature focused on optimizing the condition number under certain conditions. P. Maréchal and J. J. Ye investigated the optimization of condition number over a compact convex subset of the cone of symmetric positive semi-definite n×n matrices in 2009 [16], while X. J. Chen, R. S. Womersley, and J. J. Ye investigated the minimization of the condition number of a Gram matrix of the polynomial regression model in 2011 [17]. Both of the works introduced the idea of the Clarke generalized gradient which can accelerate the optimization process.

On the other hand, the hyper-parameters θ control the value of $K_{\sigma }\left(x\right)$; hence, one can consider the MLE (Maximum Likelihood Estimation) or MAP (Maximum A Posterior) of θ. Note that the data are sampled sequentially, which implicitly implies that the MLE (MAP) of θ satisfies the criterion with current samples, and it usually does not hold when a new point is added. Instead of using the point estimate of θ, one can consider the general technique [18,19] of integrating the acquisition function ${\bar{a}}_{K}\left(x\right)$ over the posterior distribution:

(8) $${\bar{a}}_{K}\left(x\right)=\int a_{K}\left(x\right)p\left(\theta |X,Y\right)d\theta ,$$

where p(θ|X,Y)∝p(θ)p(Y|X,θ) is the posterior distribution with the DoE (X), observations (*Y*) and prior distribution of the hyper-parameters (p(θ)). The expectation in Equation (8) generally accounts for uncertainty in the hyper-parameters or the average level of $a_{K}\left(x\right)$. ${\bar{a}}_{K}\left(x\right)$ can be approximated by the MC estimate, where the samples of θ from the posterior distribution can be obtained by the MCMC procedure. In this work, the efficient slice sampling approach proposed by I. Murray [20] was introduced to obtain samples of θ from the posterior distribution.

In fact, minimizing the condition number has more significance than generating stable inference for the GP model. In the next subsections, we show that minimizing $a_{K}\left(x\right)$ has a close connection with the prediction uncertainty as well as the information gain.

### 3.1. Connection to Optimization of the Integrated Posterior Variance

The prediction uncertainty is given as the posterior variance (v(x)) in Equation (3). We chose to integrate the posterior variance into the input domain instead of the approximation itself; the integration accounts for every point in the whole domain, and it also quantifies the uncertainty which provides the quality of the approximation. We let the input space (X) be a first-countable space equipped with a strictly Borel measure (μ), amd represented $k(x,x^{\prime })$ as a convergent series according to Mercer’s theorem [21]:

(9) $$\begin{array}{cc} & k\left(x,x^{\prime }\right)=\sum \lambda_{i}\psi_{i}\left(x\right)\psi_{i}\left(x^{\prime }\right),\\ s.t.\; & \int |k\left(x,x\right)|d\mu <\infty ,\;\sum \lambda_{i}^{2}<\infty ,\\ & \;\int \int \;k\left(x,x^{\prime }\right)g\left(x\right)g\left(x^{\prime }\right)d\mu \geq 0,\;\forall g\in L_{2}\left(X\right), \end{array}$$

where $\left\{\psi_{i}\left(x\right),i\geq 1\right\}$ forms an orthonormal basis of $L_{2}\left(X\right)$. Then, the next sample was obtained by minimizing its corresponding integrated posterior variance (IPV), i.e.,

(10) $$\begin{array}{cc} x_{\mathrm{next}} & =\arg\min_{x\in X}\int v\left(x^{\prime }\right)d\mu \left(x^{\prime }\right)\\ & =\arg\min_{x\in X}\int \left(K_{x^{\prime }x^{\prime }}-\left[K_{x^{\prime }}^{T}\;K_{{\mathrm{xx}}^{\prime }}\right]{\left[\begin{array}{cc} K_{\sigma } & K_{x}\\ K_{x}^{T} & K_{\mathrm{xx}}+\sigma^{2} \end{array}\right]}^{-1}\left[\begin{array}{c} K_{x^{\prime }}\\ K_{{\mathrm{xx}}^{\prime }} \end{array}\right]\right)d\mu \left(x^{\prime }\right)\\ & =\arg\max_{x\in X}\int \left[K_{x^{\prime }}^{T}\;K_{{\mathrm{xx}}^{\prime }}\right]{\left[\begin{array}{cc} K_{\sigma } & K_{x}\\ K_{x}^{T} & K_{\mathrm{xx}}+\sigma^{2} \end{array}\right]}^{-1}\left[\begin{array}{c} K_{x^{\prime }}\\ K_{{\mathrm{xx}}^{\prime }} \end{array}\right]d\mu \left(x^{\prime }\right)\\ & =\arg\max_{x\in X}\sum \lambda_{i}^{2}\left[\psi_{i}^{T}\;\psi_{i}\left(x\right)\right]{\left[\begin{array}{cc} K_{\sigma } & K_{x}\\ K_{x}^{T} & K_{\mathrm{xx}}+\sigma^{2} \end{array}\right]}^{-1}\left[\begin{array}{c} \psi_{i}\\ \psi_{i}\left(x\right) \end{array}\right]\\ & =\arg\max_{x\in X}\sum \lambda_{i}^{2}\alpha^{T}\left(\left[\begin{array}{cc} K_{\sigma }^{-1} & 0\\ 0^{T} & 0 \end{array}\right]+\frac{1}{v\left(x\right)+\sigma^{2}}\left[\begin{array}{cc} K_{\sigma }^{-1}K_{x}K_{x}^{T}K_{\sigma }^{-1} & -K_{\sigma }^{-1}K_{x}\\ -K_{x}^{T}K_{\sigma }^{-1} & 1 \end{array}\right]\right)\alpha \\ & =\arg\max_{x\in X}\sum \lambda_{i}^{2}\alpha^{T}\left(\left[\begin{array}{cc} K_{\sigma }^{-1} & 0\\ 0^{T} & 0 \end{array}\right]+\frac{\beta_{\sigma }\beta_{\sigma }^{T}}{v\left(x\right)+\sigma^{2}}\right)\alpha \\ & =\arg\max_{x\in X}\frac{1}{v\left(x\right)+\sigma^{2}}\sum {\left(\lambda_{i}\alpha^{T}\beta_{\sigma }\right)}^{2}, \end{array}$$

where $\alpha ={\left[\psi_{i}^{T},\psi_{i}\left(x\right)\right]}^{T}$, $\beta_{\sigma }={\left[K_{\sigma }^{-1}K_{x},-1\right]}^{T}$, $\psi_{i}={\left[\psi_{i}\left(X_{1}\right),\ldots ,\psi_{i}\left(X_{N}\right)\right]}^{T}$. The fourth equation was obtained by the orthonormality of $\phi_{i}\left(x\right)$, and we assumed that the hyper-parameters were fixed for simplicity. The last term is not easy to calculate; however, we investigated its upper bound which reflects the maximum reduction in the IPV:

(11) $$\begin{array}{cc} \frac{1}{v\left(x\right)+\sigma^{2}}\sum {\left(\lambda_{i}\alpha^{T}\beta_{\sigma }\right)}^{2} & =\frac{1}{v\left(x\right)+\sigma^{2}}\sum {\left|<\lambda_{i}\alpha ,\beta_{\sigma }>\right|}^{2}\\ & \leq \frac{1}{v\left(x\right)+\sigma^{2}}\sum <\lambda_{i}\alpha ,\lambda_{i}\alpha ><\beta_{\sigma },\beta_{\sigma }>\\ & \leq \frac{\lambda_{\max}\beta_{\sigma }^{T}\beta_{\sigma }}{v\left(x\right)+\sigma^{2}}\sum \lambda_{i}\alpha^{T}\alpha \\ & =\lambda_{\max}\frac{\beta_{\sigma }^{T}\beta_{\sigma }}{v\left(x\right)+\sigma^{2}}tr\left(K_{\sigma }\left(x\right)\right)≜{\mathrm{IPV}}_{upper}, \end{array}$$

where $\lambda_{\max}$ is the maximum $\left\{\lambda_{i}\right\}$ and tr(·) represents the trace of a matrix. The first inequality was derived by the Cauchy–Schwarz inequality, while the last equality was given with the help of Equation (9). Suppose the isotropic kernel functions, for example, the isotropic squared exponential covariance function or the isotropic Matérn covariance function, are used in the Gaussian process model, then $K_{\mathrm{xx}}$ is an invariant, as well as the term $tr(K_{\sigma }\left(x\right))$.

If we recall the SBKO criterion demonstrated in Equation (7), we have the following results:

(12) $$\begin{array}{cc} a_{K}\left(x\right) & =\kappa \left(K_{\sigma }\left(x\right)\right)=\kappa \left(K_{\sigma }{\left(x\right)}^{-1}\right)\\ & =\kappa \left(\left[\begin{array}{cc} K_{\sigma }^{-1} & 0\\ 0^{T} & 0 \end{array}\right]+\frac{\beta_{\sigma }\beta_{\sigma }^{T}}{v\left(x\right)+\sigma^{2}}\right). \end{array}$$

We let $s{\left(x\right)}_{1}\geq s{\left(x\right)}_{2}\geq \ldots \geq s{\left(x\right)}_{N+1}$ be the singular values of $K_{\sigma }\left(x\right)$, while $s_{1}\geq s_{2}\geq \ldots \geq s_{N}$ were those of $K_{\sigma }$. Note that we have the Cauchy’s interlacing theorem, which states that

(13) $$s{\left(x\right)}_{1}\geq s_{1}\geq s{\left(x\right)}_{2}\geq s_{2}\geq \ldots \geq s_{N}\geq s{\left(x\right)}_{N+1}.$$

Hence, it was derived that

(14) $$\begin{array}{cc} a_{K}\left(x\right) & =\frac{1}{s{\left(x\right)}_{N+1}}/\left[tr\left(\left[\begin{array}{cc} K_{\sigma }^{-1} & 0\\ 0^{T} & 0 \end{array}\right]+\frac{\beta_{\sigma }\beta_{\sigma }^{T}}{v\left(x\right)+\sigma^{2}}\right)-\sum_{i=2}^{N+1}\frac{1}{s{\left(x\right)}_{i}}\right]\\ & \geq \frac{1}{s_{N}}/\left[\frac{\beta_{\sigma }^{T}\beta_{\sigma }}{v\left(x\right)+\sigma^{2}}+\sum_{i=1}^{N}\frac{1}{s_{i}}-\sum_{i=2}^{N+1}\frac{1}{s{\left(x\right)}_{i}}\right]\\ & \geq \frac{1}{s_{N}}/\left[\frac{\beta_{\sigma }^{T}\beta_{\sigma }}{v\left(x\right)+\sigma^{2}}\right]. \end{array}$$

Similarly, we have

(15) $$\begin{array}{cc} a_{K}\left(x\right) & =\left[tr\left(\left[\begin{array}{cc} K_{\sigma }^{-1} & 0\\ 0^{T} & 0 \end{array}\right]+\frac{\beta_{\sigma }\beta_{\sigma }^{T}}{v\left(x\right)+\sigma^{2}}\right)-\sum_{i=1}^{N}\frac{1}{s{\left(x\right)}_{i}}\right]/\frac{1}{s{\left(x\right)}_{1}}\\ & \geq s_{1}\left[\frac{\beta_{\sigma }^{T}\beta_{\sigma }}{v\left(x\right)+\sigma^{2}}+\sum_{i=1}^{N}\frac{1}{s_{i}}-\sum_{i=1}^{N}\frac{1}{s{\left(x\right)}_{i}}\right]\\ & \geq s_{1}\left[\frac{\beta_{\sigma }^{T}\beta_{\sigma }}{v\left(x\right)+\sigma^{2}}\right]. \end{array}$$

According to Equations (14) and (15), we have the boundaries of $\beta_{\sigma }^{T}\beta_{\sigma }v\left(x\right)+\sigma^{2}$ as follows:

(16) $$\frac{\beta_{\sigma }^{T}\beta_{\sigma }}{v\left(x\right)+\sigma^{2}}\in \left[\frac{1}{s_{N}a_{K}\left(x\right)},\frac{a_{K}\left(x\right)}{s_{1}}\right].$$

By considering Equations (11) and (16) together, we obtained the lower bound of ${\mathrm{IPV}}_{upper}$ as

(17) $${\mathrm{IPV}}_{upper}=\lambda_{\max}\frac{\beta_{\sigma }^{T}\beta_{\sigma }}{v\left(x\right)+\sigma^{2}}tr\left(K_{\sigma }\left(x\right)\right)\geq \frac{\lambda_{\max}tr\left(K_{\sigma }\left(x\right)\right)}{s_{N}a_{K}\left(x\right)}.$$

The lower bound of ${\mathrm{IPV}}_{upper}$ is inversely proportional to $a_{K}\left(x\right)$, so the new sample x that minimizes the condition number also maximizes the reduction of the IPV.

### 3.2. Connection to Optimization of the KL-Divergence

Equation (7) presents a simple way to incorporate the K-optimal design and BO framework. Such a procedure ensures the success of Bayesian inference; however, it is notable that the covariance matrix (*K*) alone does not reflect how well the new sample supports the inference of model. We used Kullback–Leibler (KL) divergence [22] from the posterior to prior as a metric to illustrate the performance of the new sample, as follows:

(18) $$a_{\mathrm{KL}}\left(x\right)=-D_{\mathrm{KL}}\left(p(\theta |X,Y)∥p(\theta |X,Y,x,y)\right)=-\int p\left(\theta |X,Y\right)\log\frac{p(\theta |X,Y)}{p(\theta |X,Y,x,y)}d\theta ,$$

where p(θ|X,Y,x,y) is the posterior distribution given DoE {X,x} and a new point is sampled such that $x_{\mathrm{next}}=\arg\max_{x\in X}a_{\mathrm{KL}}\left(x\right)$. Unlike the entropy search acquisition function which maximizes the expected reduction in the negative differential entropy ($H[p\left(x_{\mathrm{best}}|X,Y\right)]$) *w.r.t* the current best location ($x_{\mathrm{best}}$), Equation (18) aims to reduce the uncertainty of the hyper-parameters, i.e., the uncertainty of the inference. We chose the inclusive direction of the KL divergence since we had p(θ|X,Y) known as the prior at each step, and the KL-divergence explicitly quantified the additional information captured in p(θ|X,Y,x,y) relative to the previous distribution, where a larger negative KL divergence reflects a greater information gain about θ upon the possible new design ({x,y}).

We note that the new observation (*y*) cannot be attained before being actually sampled at the point, so the prediction m(x) in Equation (3) is introduced to substitute the unknown *y*. However, m(x) has high uncertainty at some points; hence, Equation (18) becomes unsuitable for inference. An analogue technique is taking the expectation over the prediction which is presented as follows:

(19) $${\bar{a}}_{\mathrm{KL}}\left(x\right)=-\int p\left(\theta |X,Y\right)\left(\int p\left(f|X,Y,x,\theta \right)\log\frac{p(\theta |X,Y)}{p(\theta |X,Y,x,f)}df\right)d\theta .$$

The above acquisition function was introduced by Kim et al. [23], where ${\bar{a}}_{\mathrm{KL}}\left(x\right)$ is interpreted as the mutual information [24] between the parameter variables θ and the predictive observation f(x) (which is also a random variable given x) conditional upon candidate design x, i.e., ${\bar{a}}_{\mathrm{KL}}\left(x\right)=I\left(\theta ;f|x\right)$. Then, the next sample is obtained according to the criterion $x_{\mathrm{next}}=\arg\max_{x\in X}{\bar{a}}_{\mathrm{KL}}\left(x\right)$, i.e.,

(20) $$\begin{array}{cc} x_{\mathrm{next}} & =\arg\max_{x\in X}-\int p\left(\theta |Y\right)\left(\int p\left(f|Y,\theta \right)\left(\log p(\theta |Y)-\log p(\theta |Y,f)\right)df\right)d\theta \\ & =\arg\max_{x\in X}\int p\left(\theta |Y\right)\left(\int p\left(f|Y,\theta \right)\left(\log p(f|Y,\theta )-\log p(f|Y)\right)df\right)d\theta \\ & =\arg\max_{x\in X}H\left[E_{\theta |Y}\left(p\left(f|Y,\theta \right)\right)\right]-E_{\theta |Y}\left[H(p(f|Y,\theta ))\right]\\ & =\arg\max_{x\in X}cE_{\theta |Y}\left[H(p(f|Y,\theta ))\right] \end{array}$$

where x,X are omitted for simplicity, and H(·) represents the differential entropy. The second equation is derived from the fact that p(θ|Y) does not depend on x. Notice that H(·) is a concave function; hence, we have the last equation with a constant c>0. Now that $H\left(N\left(\mu ,\sigma^{2}\right)\right)=1/2\log\left(2\pi e\sigma^{2}\right)$, which is a strictly monotonically increasing function on $\sigma^{2}$, given Equation (3), we can rewrite Equation (20) as follows:

(21) $$x_{\mathrm{next}}=\arg\max_{x\in X}E_{\theta |Y}\left[v(x)\right].$$

The right-hand side of Equation (21) is the average uncertainty of prediction over all possible parameters (models). Specifically, we investigated v(x) only with fixed hyper-parameters (θ) for simplicity. Using Equation (16), we considered the lower bound of $a_{K}\left(x\right)$ as follows:

(22) $$a_{K}\left(x\right)\geq s_{1}\frac{\beta_{\sigma }^{T}\beta_{\sigma }}{v\left(x\right)+\sigma^{2}}\geq \frac{s_{1}}{K_{\mathrm{xx}}+\sigma^{2}}.$$

The above lower bound is an invariant if the isotropic kernel function is introduced. Since $v\left(x\right)\leq K_{\mathrm{xx}}$ (see Equation (3)), it is likely to be reached when v(x) is maximized. Hence, the minimization of $a_{K}\left(x\right)$ tends to optimize the KL-divergence between the prior and posterior distributions.

### 3.3. K-Optimal Enhanced Bayesian Optimization

Compared with the classical BO methods which aim to solve the optimization problems, the *optimization* process in the previous method focuses on the condition number of $K_{\sigma }\left(x\right)$. Actually, the DoE generated by our method tend to be scattered throughout the whole design space (the K-optimal designs are called support points in the original paper); hence, they are suitable for the global prediction behaviour of the Gaussian process model. Based on the previous discussion, the idea of K-optimality can be used to refine the classical BO methods. In this work, we focused our research on comparison with the EI criterion, which generally outperforms the PI criterion and is simpler than the LCB criterion.

The K-optimal was introduced to enhance the performance of Bayesian optimization for the following reason. It is well-known that balancing the trade-off between exploiting (where the prediction is expected to be high) and exploring (where the prediction uncertainty is high) is a key problem in the BO framework. For instance, an additional parameter, ξ, is introduced for the EI algorithm, where m(x) is replaced by m(x)+ξ in both Equations (4) and (5). The value of ξ determines the range of exploration, i.e., the anticipated improvement is likely to be greater than ξ. The choice of ξ is an open problem for researchers, and there is no universal rule to determine the optimal value of ξ. An unsuitable ξ for the EI algorithm sometimes leads to the local optimum, whose information will be strengthened as the data number increases. Notice that since the K-optimality naturally forces the samples to spread sparsely in the design space, it may be an alternative way to perform exploration.

The natural way of introducing the K-optimality to the classical BO framework is to take account of the criteria together, where we tend to choose the one that leads to a smaller condition number when several points have comparable performances in terms of the EI criterion. Given two points $x,x^{\prime }$ and corresponding classical acquisition function $a_{\mathrm{cl}}\left(\cdot \right)$, as well as $a_{K}\left(\cdot \right)$ defined in Equation (7), we have to decide which point should be sampled for four different situations considering the acquisition function and K-optimality simultaneously, which is illustrated in Table 1.

The above table shows that there two situations exist where the sample strategy remains unclear to us when combining the classical BO criteria and the K-optimality directly; hence, a new method to balance the two factors is needed. Since we aimed to solve the optimal problems in the Bayesian framework, the classical BO criteria were regarded as the main factors that indicate the direction of the next sample, while K-optimality was used to tune the strength of exploration. Basically, we have stronger belief in the point that improves the optimization results while maintaining the validity of the inference.

We used the condition number κ as the indicator of the strength of exploration. In this work, $\kappa (K_{\sigma }\left(x\right))$ was used to show the goodness of the point for the next Bayesian inference; thus, the exploration was based on the following idea: if the next point to be sampled leads to a large condition number, then we should consider extending the exploration range. We considered the analytic expression of the EI acquisition function as follows:

(23) $$\begin{array}{cc} a_{\mathrm{EI}}\left(x\right) & =(y_{\mathrm{best}}^{t}-m\left(x\right)-\xi )\Phi \left(Z\right)+v\left(x\right)\phi \left(Z\right)\\ Z & =\frac{y_{\mathrm{best}}^{t}-m\left(x\right)-\xi }{v(x)}, \end{array}$$

where ϕ(·) denotes the probability density function of the standard normal distribution. We then investigated how ξ affects the value of $a_{\mathrm{EI}}\left(x\right)$ by calculating the derivative $\partial a_{\mathrm{EI}}\left(x\right)/\partial \xi$:

(24) $$\begin{array}{cc} \frac{\partial a_{\mathrm{EI}}\left(x\right)}{\partial \xi }= & -\Phi \left(Z\right)+\left(y_{\mathrm{best}}^{t}-m\left(x\right)-\xi \right)\frac{\partial \Phi (Z)}{\partial \xi }+v\left(x\right)\frac{\partial \phi (Z)}{\partial \xi }\\ = & -\Phi (Z)-Z\phi (Z)+Z\phi (Z)=-\Phi (Z)<0. \end{array}$$

**Figure 1:**
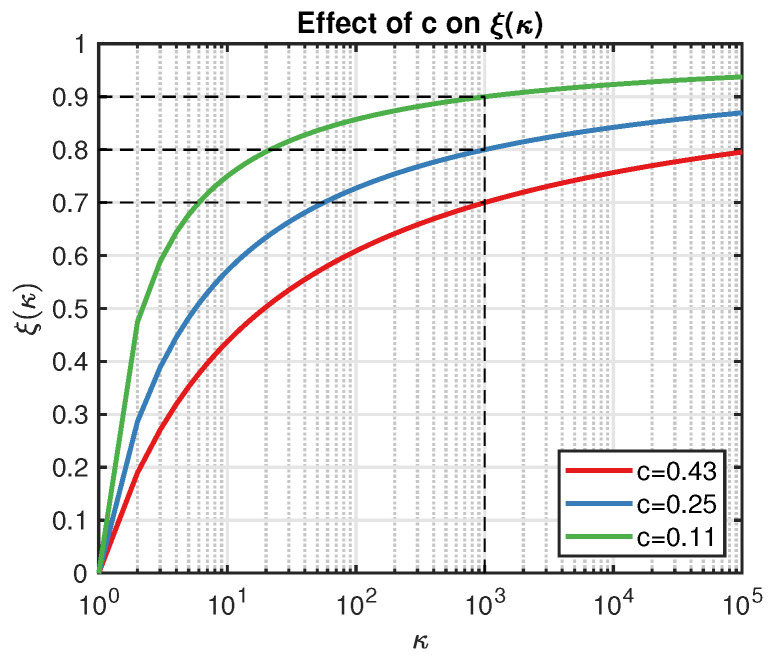
Demonstration of the effect of *c* on the shape of S(κ).

Hence, $a_{\mathrm{EI}}\left(x\right)$ is a monotonically decreasing function on ξ. Since we aimed to enlarge the utility of the point which leads to better inference (smaller condition number), the simplest way was to replace ξ with κ. However, note that the condition number κ is always greater than 1, and usually, it is a relative large number, so firstly, we normalized κ from [1,∞) to (0,1) with the help of the following function:

(25) $$\xi \left(\kappa \right)=\frac{\log\kappa }{\log\kappa +c\log\kappa_{T}},$$

where $\kappa_{T}$ is the threshold of the condition number (say, greater than 1000 as a rule of thumb), and *c* is a constant that controls the shape of ξ(κ), as illustrated in Figure 1. For example, let $\kappa_{T}=1000$ and $\xi (\kappa_{T})=0.8$; then, we have c=0.25 displayed as the blue line in Figure 1. Actually, *c* determines the exploration strength *w.r.t* $\kappa_{T}$. A smaller *c* leads to a larger ξ(κ), and we are less likely to trust the point that results in $\kappa_{T}$. On the other hand, the smaller the $\kappa_{T}$ is, the fewer the points we can accept in practice. Compared with the classical EI algorithm, ξ(κ) is more flexible because it automatically updates its value.

Several interesting features for the above methodology exist. Firstly, if a point is far away from the current exploitation region but it may result in better inference for the model, then the probability to keep it as the next sample still exists. Secondly, if a point can improve the current best value, however it may be derived from a false inference, then we are likely to dump the point by shrinking its utility. We wdemonstrate these properties in Section 4.2.

## 4. Experimental Results

The main theories and methodologies of this work are tested in this section. The first subsection demonstrates the Sequentially Bayesian K-optimal design for approximation problems, while the second one focuses on the comparison of the K-optimal enhanced Bayesian optimization problems.

### 4.1. Sequentially Bayesian K-Optimal Design for Prediction Problem

We proposed a simple acquisition function which is used to sequentially generate a DoE which ensures the validity of Bayesian inference. We consider three examples to demonstrate our SBKO method. Firstly, we implement our method on the one-dimensional Viana function [25] and the two-dimensional Branin function [26], along with comparison to alternative sampling methods. An application with the Borehole function model [27] is presented thereafter.

All of the following experiments were implemented with the Matérn 5/2 kernel, and there were three different DoE methodologies adopted for prediction comparison: the Monte-Carlo sampling strategy, the LHS method [6], and the sequential experimental design based on maximizing the posterior variance (MPV). Four measures were introduced to evaluate the performance of each method, namely, the leave-one-out cross validation error (LOO-CV), the integrated posterior variance (IPV), the root mean squared error (RMSE), and the condition number (CN). They were computed by the following formulas:

(26) $$\begin{array}{cc} \mathrm{LOO}-\mathrm{CV} & =\frac{1}{N}\sum_{i=1}^{N}{\left(m^{-i}\left(X_{i}\right)-Y_{i}\right)}^{2}\\ \mathrm{IPV} & =\int v(x)d\mu (x)\\ \mathrm{RMSE} & =\frac{1}{N^{\prime }}\sum_{i=1}^{N^{\prime }}{\left(m\left(X_{i}^{t}\right)-Y_{i}^{t}\right)}^{2}\\ \mathrm{CN} & =\kappa (K_{\sigma }), \end{array}$$

where $m^{-i}\left(\cdot \right),m\left(\cdot \right)$ represents the prediction of the GP model given {X,Y} except $\left\{X_{i},Y_{i}\right\}$, and {X,Y} respectively, while $\left\{X_{i}^{t},Y_{i}^{t},i=1,\ldots ,N^{\prime }\right\}$ was the test data set, v(x) and $K_{\sigma }$ was defined with Equation (3). The LOO-CV reflected the expected level of fit of the Gaussian process model, while the IPV estimated the overall uncertainty of prediction, and the RMSE measured the average difference between the real response and the prediction. Additionally, the CN, which we care about most in this work, showed us the robustness of Bayesian inference. Furthermore, noted that 10,000 points of independent test data were introduced to calculate the RMSE. Although, this is generally impossible for practical problems, we applied it for research purposes.

The Gaussian process models were constructed with the *gpml* toolbox [28] by Carl C. Rasmussen, and the optimizations of condition number in the SBKO were performed with the DIRECT algorithm [15] of the NLOPT library [29]. The main results were as follows:

**Example** **1.** ***Viana function** [25]*

(27) $$y=\frac{10\cos\left(2x\right)+15-5x+x^{2}}{50}+\varepsilon ,\;x∼U\left(-3,3\right),\;\varepsilon ∼N\left(0,0.01^{2}\right).$$

The number of all DoEs for Example 1 was set as 7, and specifically, a randomly sampled point was given as the initial experimental design for the SBKO and the sequential MPV design. Each of the four methods was replicated 100 times. Table 2 presents the means and standard deviations of the LOO-CV, IPV, RMSE, and CN based on 4 DoEs. It is clear that the SBKO can always lead to a smaller condition number. On the other hand, considering the LOO-CV/IPV/RMSE, the SBKO also showed the best performance with the smallest standard deviation. This means that the SBKO has the most stable performance for repeatable simulations.

**Example** **2.** ***Branin function** [26]*

(28) $$\begin{array}{cc} y= & {\left(15x_{2}-\frac{5.1}{4\pi^{2}}{\left(15x_{1}-5\right)}^{2}+\frac{5}{\pi }\left(15x_{1}-5\right)-6\right)}^{2}+10\left(1-\frac{1}{8\pi }\right)cos\left(15x_{1}-5\right)+10+\varepsilon ,\\ & x_{i}∼U\left(0,1\right),\;i=1,2,\;\varepsilon ∼N\left(0,0.01^{2}\right). \end{array}$$

We ran the experiments with similar setups for Example 2, only changing the number of DoEs to 15. The means and standard deviation of the LOO-CV, IPV, RMSE, and CN derived by 100 independent simulations are given in Table 3. It is clear that the SBKO generally outperforms the other three DoEs. The SBKO design leads to the smallest condition number; it also has the potential ability to lower the IPV, as discussed in Section 3.2. Because the MPV focuses on the point with maximum posterior variance, the experimental design tends to distribute sparsely in the whole domain which improves its global accuracy. We note that the MPV and the SBKO have comparable performances, and the reason that the SBKO is generally slightly better may be that the Bayesian inference with the SBKO is more robust than the MPV.

**Example** **3.** ***Borehole function** [27]*

The Borehole function models the flow of water through a borehole drilled from the ground surface through two aquifers. Although it is an eight-dimensional problem, it can be evaluated very fast; hence, it is commonly used test model. The explicit expression is

(29) $$y=\frac{2\pi T_{u}\left(H_{u}-H_{l}\right)}{\ln\left(r/r_{w}\right)\left(1+\frac{2LT_{u}}{\ln\left(r/r_{w}\right)r_{w}^{2}K_{w}}+\frac{T_{u}}{T_{l}}\right)}+\varepsilon ,\;\varepsilon ∼N\left(0,2^{2}\right),$$

and the input variables and their distributions are given in Table 4 as follows:

The number of the DoEs was set as 100 for the Borehole function. The comparison of the four different DoEs is given in Table 5. Obviously, the SBKO design still possesses the smallest condition number, however interestingly the LHS design generally outperforms the others *w.r.t* the LOO-CV, IPV, and RMSE. Note that the condition numbers of the four designs are approximately equal to 1, and the reason for this is that the 100 samples are located extremely sparsely in the eight-dimensional space. Since the four designs all lead to valid Bayesian inference, this limits the potential advantage of our SBKO method, such as in the Examples 1 and 2. We discussed that the SBKO design usually include points on the boundary, hence it does not distribute as evenly as the LHS design which may be the main reason that it performs a little worse than the LHS design.

The above three examples illustrate the potential usage range of the SBKO method. Basically, the SBKO outperforms the other DoEs if the required sample number leads to a compact set in the input domain where the Bayesian inference has high probability of failing. However, when we have the knowledge that those samples form a sparse set, the classical LHS design could be an option. This also reflects a potential application of the SBKO method for the high dimensional problem if we have to deal with non-linear constraints and a non-convex region where the LHS design would be inadequate.

### 4.2. K-Optimal Enhanced Bayesian Optimization Problem

In this subsection, we demonstrate the K-optimal enhanced Bayesian optimal algorithm. As discussed in Section 3.3, ξ(κ) is the indicator of the strength of exploration. We compared the classical EI algorithm (with ξ=0.01 suggested by Lizotte) and our K-optimal enhanced EI (KO-EI) algorithm. Our experiments consisted of two parts. Firstly, we illustrated the capability of exploration of the two methods. Secondly, the comparison of the convergence rate was demonstrated. The Viana function and Branin function were used as the benchmark test functions again, and the Gaussian process model was equipped with the Matérn 5/2 kernel too. We also investigated the BO algorithms on a logistic regression classification task on the MNIST data in the last experiment. The main results are as follows.

**Example** **4.** ***Comparison of Exploration***

**Figure 2:**
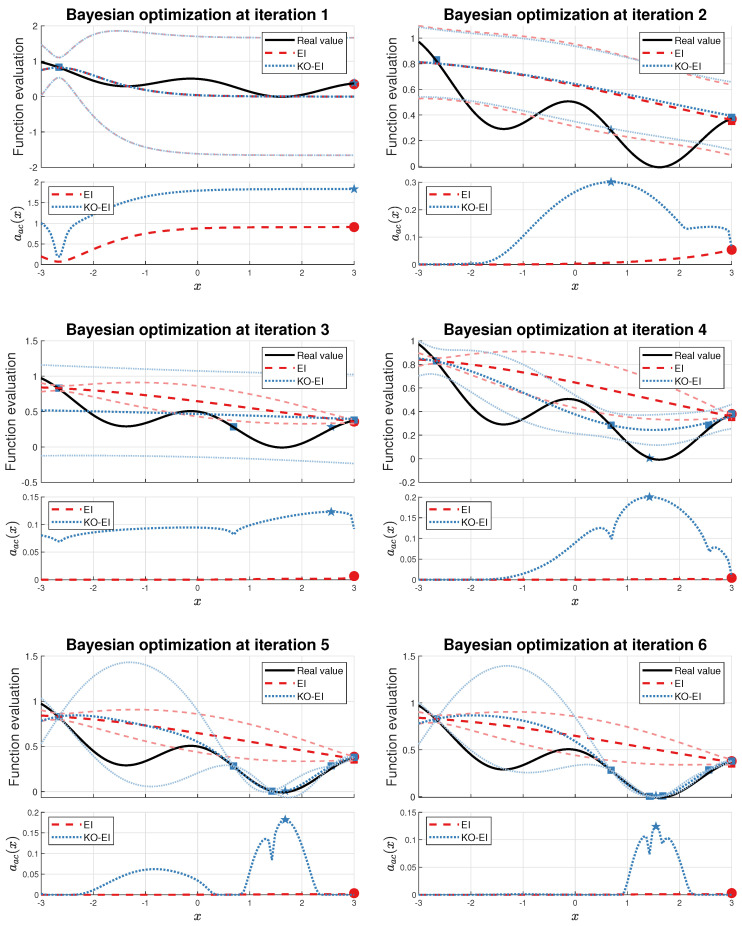
Comparisons of the expected improvement (EI) and K-optimal enhanced EI (KO-EI) algorithms. The above subfigure of each figure illustrates the predictions (darker line) and corresponding standard deviations (lighter line), and the one beloowdemonstrates the values of acquisition functions. The red and blue squares represent the current samples for the EI and KO-EI algorithm respectively. The red dots are the best points to be sampled according to the EI criterion, while the blue pentagrams were collected *w.r.t* the KO-EI algorithm.

We started our experiments with an extreme special case of the Viana function, Equation (27). We let $\left[X_{0},y_{0}\right]=\left[-2.6594,0.8303\right]$ be the initial experimental design, and then the EI and KO-EI were implemented for 6 iterations. The hyper-parameters were optimized via the maximum likelihood criterion before sampling the next point at each iteration. The corresponding values of prediction and acquisition functions are displayed in Figure 2.

It is clear that the point to maximize the EI acquisition is trapped at x=3. As the iteration continues, it strengthens the information that the corresponding Gaussian process model has ‘accurate’ approximation and the optimal value has already obtained, which is obviously a false conclusion. On the contrary, although the KO-EI sampled $X_{1}=3$ at iteration 1, it ensures that we are able to jump out the trap to reach $X_{2}=0.7008$. As discussed at the end of Section 3.3, although the point 0.7008 is far away from the current exploitation region at around x=3, we can still extract it as the next sample. Furthermore, x=3 leads to a failure in Bayesian inference, so it is eliminated from the candidate set for the next sample.

**Example** **5.** ***Comparison of Convergence Rate***

**Figure 3:**
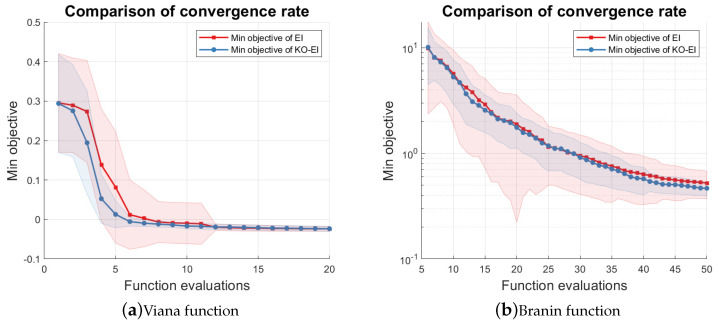
Comparison of convergence rate between the EI and KO-EI algorithms. The solid dash line represents the mean value of the minimal observed objective, while the transparent region represents the standard deviation *w.r.t* 100 independent simulations. (**a**) Viana function; (**b**) Branin function.

In this part, we started a new experiment to investigate the convergence rate of the two algorithms with the Viana and Branin function, Equation (28). We repeated the EI and KO-EI optimization 100 times. The numbers of initial design for the Viana function and the Branin function were set to be 1 and 5, respectively, while the maximum numbers of samples were set to be 20 and 50, respectively. The results are summarized and displayed in Figure 3. Figure 3a shows that the KO-EI convergence is faster than the EI criterion for the Viana function, and generally, the KO-EI is more stable than the EI algorithm, because the standard deviation of minimal observed objective is always smaller. When applying the two algorithms with the Branin function, the two methods have similar convergence rates and the KO-EI criterion is slightly better. However, we note that the KO-EI has a smaller standard deviation of the minimal observed objective again; hence, it can lead to a more stable result.

**Example** **6.** ***Application on the Logistic Regression Classification Task***

**Figure 4:**
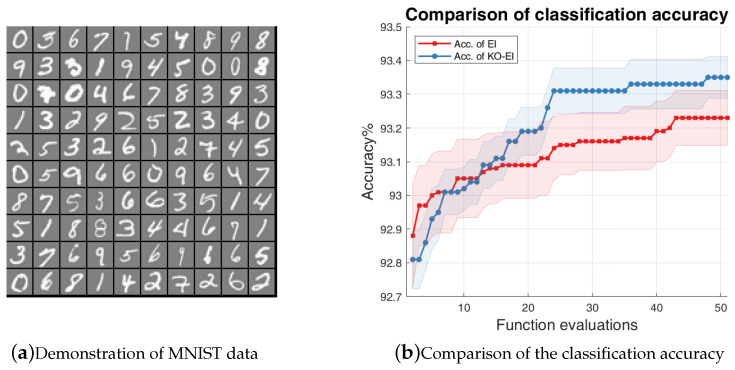
Comparison of accuracy between the EI and KO-EI algorithms on the logistic regression classification on MNIST data. (**a**) Demonstration of the MNIST data; (**b**) The solid dash line represents the mean value of the classification accuracies while the transparent region represents the standard deviation *w.r.t* 50 independent simulations.

We used the EI and KO-EI algorithms to optimize the hyper-parameters of the logistic regression algorithms to maximize the classification accuracy. The experiment was implemented with the MNIST data, which was also investigated in reference [18]. The hyper-parameters of the logistic regression classification algorithm were the $L^{2}$ regularization parameter λ, between 0 and 1; the constants σ, ρ of the Wolfe–Powell conditions with σ∈[0,0.5] and ρ∈[0,1], respectively; the batch size from 10 to 100; the number of learning iterations from 20 to 200; and the learning slope ratio between 5 and 15. We compared the classification accuracies for 50 independent simulations for 50 EI/KO-EI runs, and the results are given in Figure 4. Note that the y-axis of Figure 4b is the percentage of classification accuracy times 100. It is obvious that the KO-EI performs better after several rounds of optimization, and it also leads to smaller standard deviations.

## 5. Conclusions

This paper examined the combination of the K-optimal design and the Bayesian optimization framework. In order to ensure the validity of Gaussian process inference, we introduced the condition number of the Gram matrix of the kernel as the acquisition function to propose the Sequentially Bayesian K-optimal design (SBKO). The SBKO is suitable for global tasks, such as approximation and prediction. On the other hand, the property of K-optimality was also used with the classical BO methods, namely the KO-BO method, in this research. The trade-off parameters were updated automatically based on the idea that points leading to smaller condition numbers should be explored. Numerical investigations on the approximation problem results showed that the SBKO generally outperforms the MC, LHS, and the MPV when the samples are compact in the input domain. Examples on the optimization problem showed that the K-optimal enhanced expected improvement (KO-EI) can deal with extreme cases where the EI criterion is trapped in a local maximum very well. Further experiments showed that the KO-EI convergences faster than the EI algorithm; however, it is much more stable.

Although the K-optimality performed well in our experiments, we also note that its calculation and optimization could still be a burden because it is not convex and there is no explicit expression of its gradient. Future work could focus on the approximation methods of the condition number, such as the Clarke generalized gradient [16,17], hence accelerating corresponding computation. We could also investigate the usage of our method to deal with non-convex constraints and input domains. An analysis of the theoretical boundaries of the KO-BO algorithms would be of great interest too.

## Figures and Tables

**Table 1:** Four situations considering the acquisition function and K-optimality simultaneously.

	$a_{\mathrm{cl}}\left(x\right)<a_{\mathrm{cl}}\left(x^{\prime }\right)$	$a_{\mathrm{cl}}\left(x\right)\geq a_{\mathrm{cl}}\left(x^{\prime }\right)$
$a_{K}\left(x\right)<a_{K}\left(x^{\prime }\right)$	**Not decided**	x
$a_{K}\left(x\right)\geq a_{K}\left(x^{\prime }\right)$	$x^{\prime }$	**Not decided**

**Table 2:** Means and standard deviations of the leave-one-out cross validation error (LOO-CV), the integrated posterior variance (IPV), the root mean squared error (RMSE) and the condition number (CN) based on 4 DoEs in Example 1, where the number in the parentheses is the standard deviation and the bold numbers represent the best outcomes.

	LOO-CV	IPV	RMSE	CN
MC	**0.5528** (0.3874)	0.0778 (0.1100)	0.1644 (0.1734)	1.2651 × 10${}^{5}$ (8.4871 × 10${}^{5}$)
LHS	0.7557 (0.2217)	0.0566 (0.1032)	0.0837 (0.1354)	3.2924 (0.9513)
MPV	0.6242 (0.1916)	0.0104 (0.0044)	0.0254 (0.0078)	2.5248 (0.5519)
BKO	0.6771 (**0.1711**)	**0.0093** (**0.0025**)	**0.0241** (**0.0072**)	**2.2601** (**0.4658**)

**Table 3:** Means and standard deviations of the LOO-CV, IPV, RMSE, and CN based on 4 DoEs in Example 2 where the number in the parentheses is the standard deviation. Bold numbers represent the best outcomes.

	LOO-CV	IPV	RMSE	CN
MC	0.1832 (0.1416)	300.0422 (219.0879)	23.0570 (8.8187)	7.4361 × 10${}^{4}$ (2.2117 × 10${}^{5}$)
LHS	0.3032 (0.2120)	216.2945 (223.1399)	20.6949 (9.0519)	1.5387 × 10${}^{3}$ (645.5456)
MPV	0.1825 (0.1806)	109.4216 (103.9399)	12.8602 (**2.6683**)	195.8292 (27.0753)
BKO	**0.1553** (**0.1114**)	**97.4508** (**42.3682**)	**12.2822** (2.8295)	**113.3958** (**20.5164**)

**Table 4:** The input variables of the Borehole function and their usual distributions.

	Input	Distribution	Unit
$r_{w}$	radius of borehole	U(0.05,0.15)	m
*r*	radius of influence	U(100,50,000)	m
$T_{u}$	transmissivity of upper aquifer	U(63,070,115,600)	m${}^{2}$/yr
$H_{u}$	potentiometric head of upper aquifer	U(990,1110)	m
$T_{l}$	transmissivity of lower aquifer	U(63.1,116)	m${}^{2}$/yr
$H_{l}$	potentiometric head of lower aquifer	U(700,820)	m
*L*	length of borehole	U(1120,1680)	m
$K_{w}$	hydraulic conductivity of borehole	U(9855,12,045)	m/yr

**Table 5:** Means and standard deviations of the LOO-CV, IPV, RMSE, and CN based on 4 DoEs in Example 3, where the numbers in parentheses are the standard deviations and the bold numbers are the best results.

	LOO-CV	IPV	RMSE	CN
MC	0.0024 (0.0012)	8.5170 (1.0624)	2.0212 (0.3855)	1 + 1.0808 × 10${}^{-6}$ (2.9431 × 10${}^{-7}$)
LHS	**0.0024** (**0.0010**)	**8.0039** (0.7151)	**1.9091** (**0.2877**)	1 + 1.0808 × 10${}^{-6}$ (2.9431 × 10${}^{-7}$)
MPV	0.0033 (0.0028)	11.2356 (2.8431)	2.7163 (0.5043)	1 + 6.3612 × 10${}^{-7}$ (2.6469 × 10${}^{-7}$)
BKO	0.0026 (0.0013)	8.8565 (**0.4842**)	2.2822 (0.2895)	**1 + 5.6624 × 10${}^{-8}$** (**1.0439 × 10${}^{-7}$**)
